# Supplementary material for: Biomarker Profiling of Upper Tract Urothelial Carcinoma Only and with Synchronous or Metachronous Bladder Cancer
Source: Biomedicines. 2024 Sep 23;12(9):2154. doi: 10.3390/biomedicines12092154 (PMC11429062; doi:10.3390/biomedicines12092154)
Supplement: Supplementary file 1 [file biomedicines-12-02154-s001.zip › biomedicines-3167613-supplementary.pdf]

biomedicines

MDPI

Supplementary Materials: Biomarker Profiling of Upper Tract Urothelial Carcinoma Only and with Synchronous or Metachronous Bladder Cancer

Sara Meireles, Carolina Dias, Diana Martins, Ana Marques, Nuno Dias and Luís Pacheco-Figueiredo, João Silva, Carlos Martins Silva, Miguel Barbosa, Luís Costa, José Manuel Lopes and Paula Soares

Table S1. Correlation between p53 IHC expression and *TP53* alteration status.

***TP53* alteration status**	**p53 IHC**		**Total**	***p value***
	Wild type pattern	Aberrant type pattern		
Wild type	15 (75)	13 (65)	28 (70)	0.490
Mutated	5 (25)	7 (35)	12 (30)	
**Total**	20 (50)	20 (50)	40	

Abbreviations: IHC – immunohistochemical.

Table S2. Association of clinicopathologic features with IHC markers and genes expression in patients with UTUC.

Abbreviations: AJCC – American Joint Committee on Cancer; CK – cytokeratin; cm - centimeter; ECOG PS – Eastern Cooperative Oncology Group Performance Status; IHC – immunohistochemical; LVI – lymphovascular invasion; n – number of patients; UBC – urothelial bladder cancer; UC – urothelial carcinoma; UTUC – upper tract urothelial carcinoma.

Characteristics	CK5/6	*p value*	CK20	*p value*	GATA3	*p value*	p53	*p value*	*FGFR3*	*p value*	*TERT*	*p value*	*RAS*	*p value*
**Smoking**														
Yes	28 (63.6)	.108	24 (58.5)	.542	21 (56.8)	.786	10 (47.6)	.433	25 (61)	.299	27 (60)	.404	4 (57.1)	.613[a]
No	16 (36.4)		17 (41.5)		16 (43.2)		11 (52.4)		16 (39)		18 (40)		3 (42.9)	
**Hydronephrosis**														
Yes	26 (49.1)	.849	29 (53.7)	.394	30 (57.7)	.107	19 (65.5)	.051	32 (60.4)	.056	29 (50.9)	.928	5(62.5)	.489[a]
No	27 (50.9)		25 (46.3)		22 (42.3)		10 (34.5)		21 (39.6)		28 (49.1)		3(37.5)	
**Tumor location**														
Renal pelvis	32 (59.3)	.337	29 (51.8)		24 (45.3)	.059	14 (48.3)	.505[a]	31 (56.4)	.534	39 (67.2)	.087	7 (77.8)	.392[a]
Ureter	17 (31.5)		18 (32.1)	.537	20 (37.7)		10 (34.5)		18 (32.7)		11 (19)		1 (11.1)	
Both	5 (9.3)		9 (16.1)		9 (17)		5 (17.2)		6 (10.9)		8 (13.8)		1 (11.1)	
**Histological subtype**														
Pure UC	45 (83.3)	.046	51 (91.1)	.585	51 (96.2)	.029	25 (86.2)	.501[b]	50 (90.9)	.608	53 (91.4)	.416	4 (44.4)	.001
Non-pure UC	9 (16.7)		5 (8.9)		2 (3.8)		4 (13.8)		5 (9.1)		5 (8.6)		5 (55.6)	
**Tumor size, cm**														
≤ 2	6 (11.3)	.542	10 (17.9)	.264	10 (19.2)	.153	6 (20.7)	.209[a]	4 (7.3)	.060	4 (6.9)	.048	1 (11.1)	.627
> 2	47 (88.7)		46 (82.1)		42 (80.8)		23 (79.3)		51 (92.7)		54 (93.1)		8 (88.9)	
**Multifocality**														
Yes	7 (13)	.268	7 (12.5)	.311	4 (7.5)	.479	1 (3.4)	.284[a]	7 (12.7)	.296	5 (8.6)	.611	0 (0)	.597[a]
No	47 (87)		49 (87.5)		49 (92.5)		28 (96.6)		48 (87.3)		53 (91.4)		9 (100)	
**Tumor grade**														
Low-grade	3 (5.8)	.571[a]	5 (8.9)	.438[a]	2 (3.9)	.452[a]	2 (6.9)	.589[a]	3 (5.5)	.652[a]	3 (5.2)	.588[a]	0 (0)	.547[a]
High-grade	49 (94.2)		51 (91.1)		49 (96.1)		27 (93.1)		52 (94.5)		55 (94.8)		9 (100)	
**LVI**														
Yes	33 (66.7)	.693	12 (21.8)	.006	15 (30)	.427	14 (48.3)	.070	12 (22.2)	.008	19 (33.3)	.840	3 (33.3)	.631[a]
No	19 (33.3)		43 (78.2)		35 (70)		15 (51.7)		42 (77.8)		38 (66.7)		6 (66.7)	
**Carcinoma in situ**														
Yes	7 (13)	.207	6 (10.7)	.060	12 (22.6)	.182	6 (20.7)	.625	5 (9.1)	.002	8 (13.8)	.308	0 (0)	.356[a]
No	47 (87)		50 (89.3)		41 (77.4)		23 (79.3)		50 (90.9)		50 (86.2)		9 (100)	
**AJCC staging**														
0is + 0a + I	17 (31.5)	.651	22 (40)	.025	16 (30.2)	.983	2 (7.1)	.003	19 (34.5)	.314	18 (31)	.803	2 (22.2)	.721[a]
II + III + IV	37 (68.5)		33 (60)		37 (69.8)		26 (92.9)		36 (65.5)		40 (69)		7 (77.8)	
**Lymph node involvement**														
Yes	6 (35.3)	.407[a]	1 (7.7)	.047[a]	2 (25)	.645[a]	2 (28.6)	.623[a]	5 (27.8)	.604[a]	5 (27.8)	.604[a]	2 (50)	.284
No	11 (64.7)		12 (92.3)		6 (75)		5 (71.4)		13 (72.2)		13 (72.2)		2 (50)	
**Metastasis at diagnosis**														
Yes	6 (11.1)	0.90	3 (5.4)	.046	5 (9.4)	.537	3 (10.3)	.820	6 (10.9)	.847	7 (12.1)	.931	0 (0)	.595[a]
No	48 (88.9)		53 (94.6)		48 (90.6)		26 (89.7)		49 (89.1)		51 (87.9)		9 (100)	

Figure S1. Kaplan Meier curves for progression-free survival (PFS) (**A**) and overall survival (OS) (**B**) in luminal and basal subtypes of entire UTUC cohort.

A

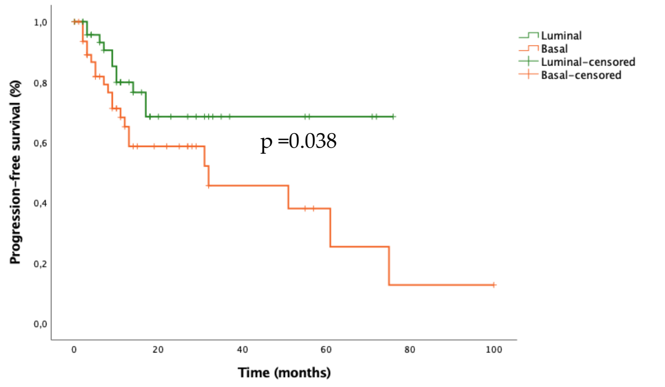

B.

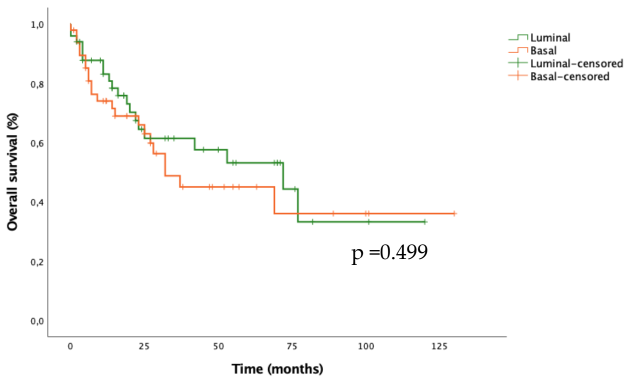

Log-rank test, statistical significance p value < 0.05

Figure S2. Kaplan Meier curves for progression-free survival (PFS) (**A**) and overall survival (OS) (**B**) in UTUC patients according to *FGFR3*, *TERT* and *RAS* status.

A

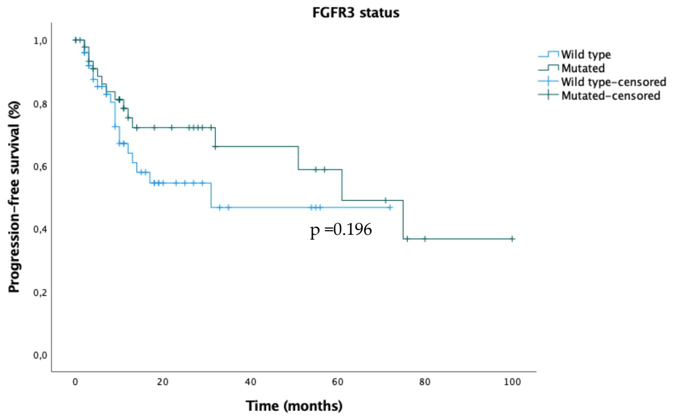

B.

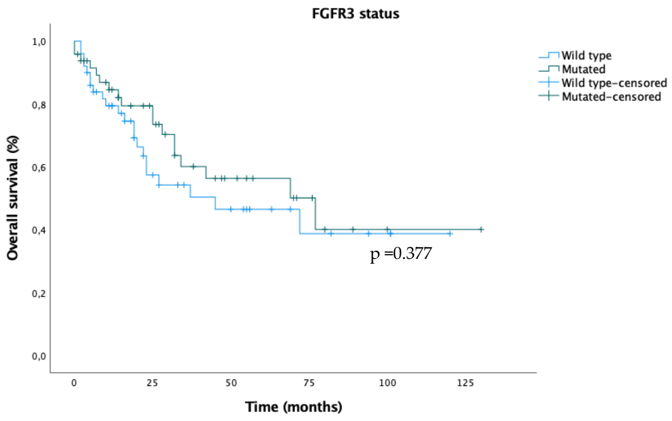

Log-rank test, statistical significance p value < 0.05

A

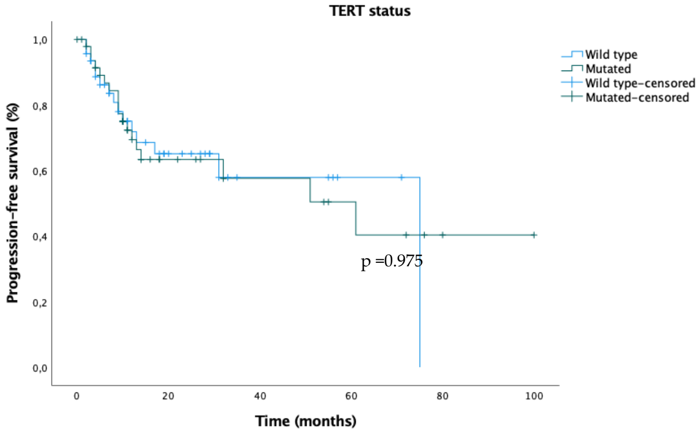

B.

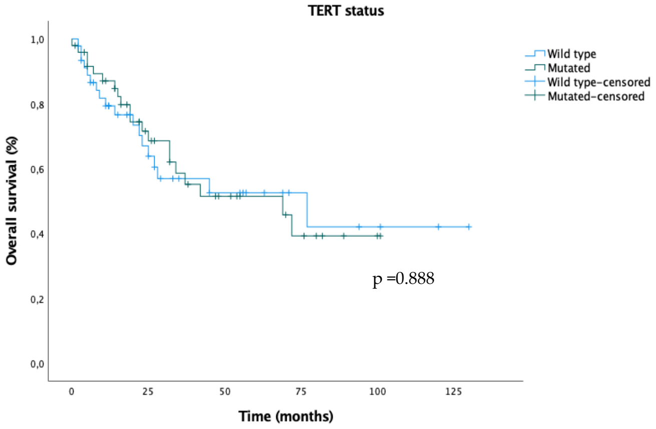

Log-rank test, statistical significance p value < 0.05

A

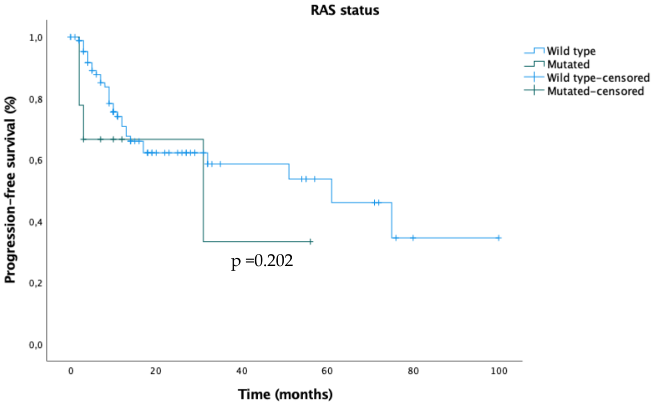

B.

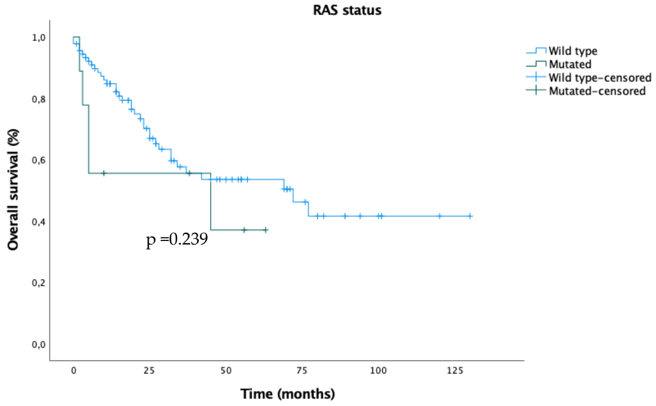

Log-rank test, statistical significance p value < 0.05
